# Negative Influence of a Long-Term High-Fat Diet on Murine Bone Architecture

**DOI:** 10.1155/2014/318924

**Published:** 2014-02-20

**Authors:** Hinrich Fehrendt, Thomas Linn, Sonja Hartmann, Gabor Szalay, Christian Heiss, Reinhard Schnettler, Katrin Susanne Lips

**Affiliations:** ^1^Laboratory for Experimental Trauma Surgery, Justus Liebig University Giessen, Kerkraderstrasse 9, 35394 Giessen, Germany; ^2^Clinical Research Unit, Medical Clinic and Polyclinic 3, Justus Liebig University Giessen, Klinikstrasse 33, 35392 Giessen, Germany; ^3^Department of Trauma Surgery, University Hospital of Giessen-Marburg, Rudolf-Buchheim-Strasse 7, 35392 Giessen, Germany

## Abstract

A correlation between obesity and bone metabolism is strongly assumed because adipocytes and osteoblasts originate from the same precursor cells and their differentiation is conversely regulated by the same factors. It is controversially discussed if obesity protects bone or leads to loss of bone mass. Thus, the aim of the present study was to investigate the influence of diet-induced mild obesity (11% increased body weight compared to control) on bone microstructure in mice. Four-week-old male C57BL/6J mice received a high-fat diet (HFD, 60% kcal from fat) and were analyzed by means of dual X-ray absorptiometry, histological methods, real-time RT-PCR, and transmission electron microscopy in comparison to control animals (10% kcal from fat). The cancellous bone mass, collagen 1*α*1 expression, amount of osteoid, and cohesion of cells via cell-to-cell contacts decreased in HFD mice whereas the bone mineral density and the amount of osteoblasts and osteoclasts were not modified. The amount of apoptotic osteocytes was increased in HFD mice in comparison to controls. We conclude that moderately increased body weight does not protect bone architecture from age-dependent degeneration. By contrast, bone microstructure is negatively affected and reduced maintenance of cell-cell contacts may be one of the underlying mechanisms.

## 1. Introduction

Obesity is characterized by a body-mass index of ≥30 kg/m² and excessive body fat accumulation [1]. Globally, estimated 502 million adults and 170 million children were classified as overweight or obese in 2008 and the rates of obesity are still increasing [2]. Obesity is associated with many chronic disorders such as type 2 diabetes mellitus, coronary heart disease, sleep-breathing disorders, certain cancers, and osteoarthritis [1]. A correlation between obesity and bone metabolism is strongly assumed. Bone forming osteoblasts are derived from stem cells that can also give rise to adipocytes and chondrocytes [3]. Stem cells proliferate and differentiate into preosteoblasts and finally into mature osteoblasts that are characterized by cessation of cell division, production of bone matrix, and synthesis of several essential marker enzymes and proteins for bone formation, for example, collagen type 1, osteocalcin, and alkaline phosphatase (ALP). Collagen type 1 is with approximately 90% of the main component of the bone matrix. In lamellar bone, its fiber organization allows the highest density per unit volume of tissue. In the biomechanically weaker woven bone collagen fibers are not so tightly packed and found in randomly oriented bundles. Crystals of hydroxyapatite are situated on the collagen fibers, within them, and in the matrix around them. They tend to be oriented in the same direction as the collagen fibers. ALP is the main enzyme for formation of hydroxyapatite crystals and is therefore important for mineralization of bone and bone mineral density (BMD). Besides the collagens several non-collagenous proteins are present in the bone matrix. Osteocalcin is the major non-collagenous protein. It is produced by osteoblasts, makes up 1% of the matrix, and plays a role in calcium binding and stabilization of hydroxyapatite in the matrix [4]. Factors stimulating formation of bone are inhibiting adipogenesis and vice versa [3]. For example, mechanical loading promotes osteoblastogenesis and inhibits differentiation of stem cells into adipocytes by increasing stable beta-catenin and reducing peroxisome proliferator-activated receptor-gamma (PPAR*γ*) whereas stimulation of PPAR*γ* decreases osteoblast differentiation and enhances adipogenesis [5–10]. However, the loss of bone mass determined in aging, osteoporosis, and after administration of glucocorticoids is associated with an increase in bone marrow adipogenesis (reviewed in [10]). The underling mechanisms for these effects are still unknown. The involvement of adipocyte-derived proinflammatory cytokines and hormones is discussed [10–12]. Since several studies focused on obesity associated with low-grade chronic inflammation, there is an increasing amount of reports describing detrimental effects of excessive body fat on bone [13–15]. There is a higher incidence of clinical fractures in obese postmenopausal women [16] and in overweight adolescents [17–19]. Several animal studies supported this negative effect on bone strength [20, 21], bone mineral density [22], and bone formation [15]. However, the traditional view of obesity is that overweight is beneficial to bone [11, 23–25] since the femoral neck of obese women especially with osteoporotic bones showed a reduction in fracture risk [25] and an increase in BMD [23]. The enhanced BMD on the weight-bearing site implies that the mechanical effect of overweight stimulates bone mineralization [24] in addition to upregulation of bone formation enhancing molecules, for example, adipocytic estrogens [26]. However, fat and bone are linked by multiple pathways. Thus, we analyzed in the present study the bone density, the cellular structures, and the molecular bone markers. Since Cao et al., 2010, [27] reported detrimental effects on bone in a murine obesity model with high-fat diet (HFD) determined by means of *μ*CT experiments, we hypothesized that our investigation will also point out negative effects of obesity on bone properties using several cell biological methods for investigation of in vivo bone samples. The expected results will gain new insights into the mechanism underling alterations of bone structure by obesity.

## 2. Materials and Methods

### 2.1. Animals and Experimental Model

All animal procedures were approved by the local ethics committee (GI20/11-Nr. A17/2010) and conducted in accordance to the Declaration of Helsinki. Seven 4-week-old male C57BL/6J mice (Janvier, France) were fed for 23 weeks with a high-fat diet (Altromin, Lage, Germany, 60% kcal from fat) and six with normal diet (Altromin, 10% kcal from fat). The animals were kept under a 12-hour (h) light and dark cycle and had free access to chow and water. The body weight was measured every week. At the age of 27 weeks the animals were euthanized by inhalation of CO_2_ and directly scanned via Dual-X-Ray Absorptiometry (DXA, lunar prodigy, GE Healthcare, Munich, Germany) for determination of bone mineral density (BMD). Afterwards bones were extracted and used for conduction of cell and molecular-biological methods.

### 2.2. Histology

Samples of femur and vertebrae L2 were fixed in 4% phosphate buffered paraformaldehyde (Carl Roth, Karlsruhe, Germany) and demineralized in 10% ethylene diamine tetra acetic acid (Merck, Darmstadt, Germany) in 0.281 M Tris-buffer. The samples were dehydrated using increasing ethanol concentrations (70%, 80%, and 96% each for 2 h, 3x 100% for 3 h) and incubated in xylol (Carl Roth, 3x 1 h) and afterwards in liquid paraffin. After blocking out, paraffin sections were cut with a thickness of 4-5 *μ*m at the rotation microtome (RM 2155, Leica, Bensheim, Germany). Sections were stained with hematoxylin and eosin (HE, Merck) or used for enzyme- or immunohistochemistry (IHC).

### 2.3. Tartrate-Resistant Acidic Phosphatase (TRAP)

To detect macrophages and osteoclasts the tartrate-resistant acidic phosphatase (TRAP) enzyme histochemistry was performed. Therefore paraffin sections were deparaffinized with xylol and a decreasing series of ethanols. After washing in 0.1 M acetate buffer (pH 5.2) sections were incubated in a solution of naphthol-AS-TR-phosphate (Sigma-Aldrich, Steinheim, Germany), di-sodium-tartrate-dihydrid (Merck), and fast red TR salt (Sigma-Aldrich) in acetate buffer at 37°C for 30 minutes (min). After washing in aqua dest., sections were counterstained with hematoxylin and coverslipped with Kaisers Glyceringelatine (Merck).

### 2.4. Alkaline Phosphatase (ALP)

The rehydrated paraffin sections were incubated in a solution of 5-bromo-4-chloro-indolyl-phosphate (BCIP) and nitro blue tetrazolium (NBT, KPL, Gaithersburg, MD, USA) salt for 45 min in a moist chamber at 37°C. After thoroughly washing in aqua dest., sections were counterstained with nuclear fast red, dehydrated, and coverslipped with DePex (Serva, Heidelberg, Germany).

### 2.5. Immunohistochemistry

Immunohistochemical incubation with an antibody for detection of collagen-1 was conducted in the present study to evaluate bone architecture. Therefore rehydrated paraffin sections were treated with a Tris-NaCl buffer (TBS, pH 7.4) containing 0.025% Triton-X-100 (Merck). Afterwards the endogenous peroxidase was blocked by incubation with 3% H_2_O_2_. After washing in TBS, the sections were incubated with the primary collagen-1 antibody (Biomex, Heidelberg, Germany) in a dilution of 1 : 50 in diluting buffer (Dako, Hamburg, Germany) overnight at 4°C. After washing in TBS, sections were incubated for 30 min with a biotinylated goat anti-rabbit secondary antibody (dilution 1 : 500; Vector Laboratories Inc., Burlingame, CA, USA) and subsequently with the streptavidin-biotin-peroxidase complex (Vector Laboratories Inc.). To visualize the peroxidase a Nova-Red substrate kit (Vector Laboratories Inc.) was used and according to the manufacturer's protocol with an incubation time of 5 min. The nuclei were counterstained with hematoxylin and the sections were coverslipped with DePex. As negative control the procedure was performed omitting the first antibody.

All labeled paraffin sections were evaluated light microscopically with a photomicroscope (Axiophot-2, Zeiss, Jena, Germany) equipped with a digital camera (DC 500, Leica, Bensheim, Germany).

### 2.6. Bone Histomorphometry

For quantification of two dimensional trabecular regions in relation to the whole tissue histomorphometrical analyses were performed according to the methods described by Dempster et al., 2013, [28]. In brief, an area of interest from the tissue was defined (in mm^2^) in which the trabeculae were marked and calculated in mm^2^ with the Image-Pro Plus Software (Media Cybernetic, Maryland, USA). Afterwards the relation was calculated in percentage. As area of interest the metaphyseal region of the proximal and distal femur and the spongious part of the corpus vertebrae were used.

### 2.7. Real-Time RT-PCR

For expression analyses humeri and vertebrae L3 were collected in RNA-later (Ambion Applied Biosystems, Foster City, CA, USA), homogenized with a mortar, and transferred into 1 mL Trizol (Invitrogen, Darmstadt, Germany). After 5 min 200 *μ*L chloroform (Sigma-Aldrich) was added, the samples were centrifuged (12,000 g, 15 min, 4°C), and the upper phase containing the RNA was transferred into a new cup. Isopropanol (0.5 mL) was added and total RNA precipitated by centrifugation (12,000 g, 10 min, 4°C).

For reverse transcription of total RNA the Quantitect Reverse Transcription Kit (Qiagen, Hilden, Germany) was used. In brief, 1 *μ*g RNA was cleaned from genomic DNA by incubation with 2 *μ*L DNA Wipeout buffer for 2 min at 42°C. Afterwards RNA was transcribed to cDNA with 1 *μ*L Quantiscript reverse transcriptase, 4 *μ*L buffer, and 1 *μ*L primer mix containing Oligo(dT)s and random-primers at 42°C for 30 min. Reverse transcriptase was inactivated at 95°C for 3 min. Subsequent real-time RT-PCR was performed in the Lightcycler (Roche, Rotkreuz, Schweiz). Therefore 2 *μ*L of cDNA was added to 2 *μ*L Roche reagent (LightCyclerFastStart DNA Maser SYBR Green I, Roche, Mannheim, Germany), 0.2 *μ*L forward and reverse primer (Eurofins MWG Operon, Ebersberg, Germany, Table 1), and 6.8 *μ*L RNase free water. The samples were incubated for 10 min at 95°C, followed by 40 cycles of 5 seconds (sec) heating at 95°C, annealing for 5 sec at 58–62°C, and elongating for 5 sec at 72°C. Subsequently the PCR product was controlled by melting curve and gel electrophoresis. As controls RT negative controls were performed where the reverse transcription was conducted without the enzyme. In addition negative controls were made where the template was omitted and water was used instead (water control). Using the Lightcycler software Cp-values were measured, normalized to the reference gene *β*-actin and the ΔΔCp, and relative expression was calculated according the ΔΔCp method.

### 2.8. Transmission Electron Microscopy (TEM)

Vertebrae L4 were fixed in yellow fix (2% paraformaldehyde, 2% glutardialdehyde and 0.02% picrinic acid in 0.01 M phosphate buffer, pH 7.2) for 6 h. After washing in 0.1 M cacodylate buffer samples were incubated for 2 h in 1% osmium tetroxide solution. Afterwards they were dehydrated through an increasing ethanol series, washed 3x in xylol, and incubated in a solution of xylol and Epon (Serva). Finally the samples were polymerized in Epon and cut into semi-thin (500 nm) and ultra-thin sections (60–80 nm). The semi-thin sections were stained with toluidine blue and safranin-O and evaluated with a light microscope. The ultra-thin sections were contrasted with uranyl acetate and lead citrate and analyzed with a TEM (LEO EM 912, Zeiss, Oberkochen, Germany) equipped with a CCD-Camera (Olympus, Münster, Germany).

### 2.9. Statistical Analysis

The SPSS software (version 21.0; SPSS Institute Inc, Chicago, IL, USA) was used for statistical analysis. Comparisons were performed by the Mann-Whitney test. A *P* value of less than 0.05 indicates a significant difference.

## 3. Results

### 3.1. Body Composition

**Figure 1:**
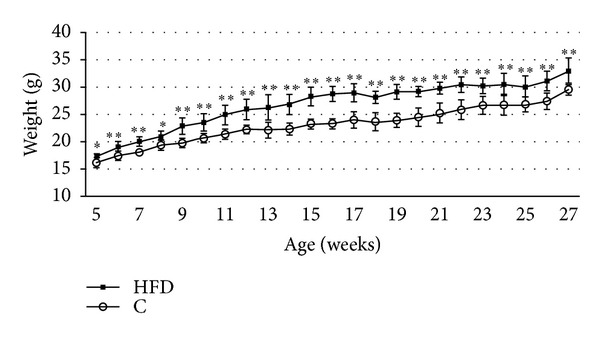
Body composition. The body weight was significantly upregulated in the high-fat diet (HFD, 60% kcal from fat) mice (*n* = 7) compared to control (C) mice (*n* = 6). Significant data were labeled with *P ≤ 0.05 and **P ≤ 0.01. Data are shown as mean ± SD.

Body weight was recorded weekly. During the experimental period of 23 weeks the weight of mice receiving a HFD (60% kcal of energy from fat) was 11% higher (*P* = 0.001) than that of control animals (10% kcal of energy from fat) (Figure 1).

### 3.2. Bone Mineral Density

**Figure 2:**
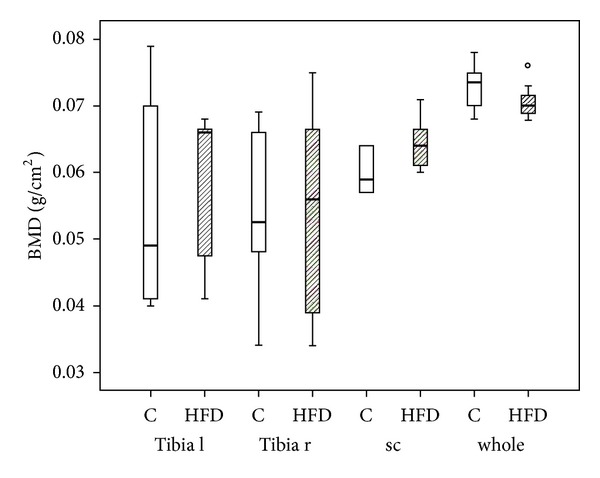
Bone mineral density. DXA measurement revealed no significant differences in bone mineral density (BMD) between the mice receiving high-fat diet (HFD; *n* = 7) and the controls (C; *n* = 6). Data are presented as box plot with the median indicated by solid line within the box. The circle represents data beyond 1.5x the interquartile range of the median. l = left, r = right, sc = spinal column, and whole = whole body.

DXA-scan did not reveal significant differences in bone mineral density between both experimental groups (Figure 2).

### 3.3. Light Microscopic Observations

**Figure 3:**
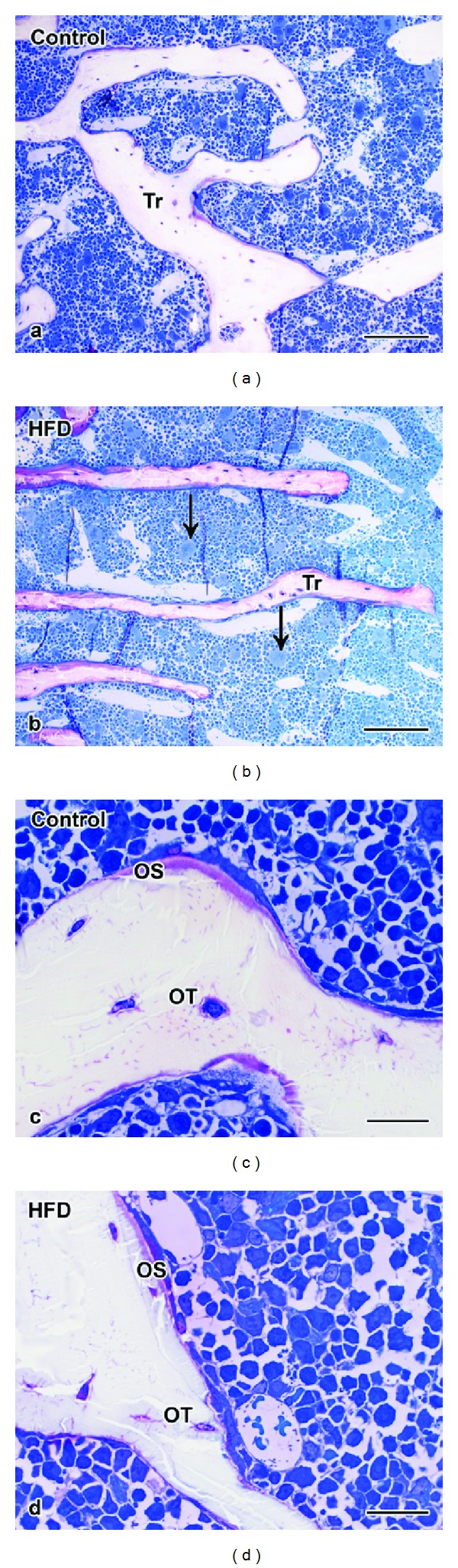
Microstructure of cancellous bone in mineralized semi-thin sections. The trabeculae of (a) control mice were wider and more connected than the trabeculae of (b) HFD mice. Higher magnification revealed that (c) control mice dispose more osteoid than (d) HFD mice. Tr = trabculae, OS = osteoid, OT = osteocyte, C = control mice, and arrow = megakaryocytes. Scale bar: 100 *μ*m in ((a)-(b)) and 20 *μ*m in ((c)-(d)).

**Figure 4:**
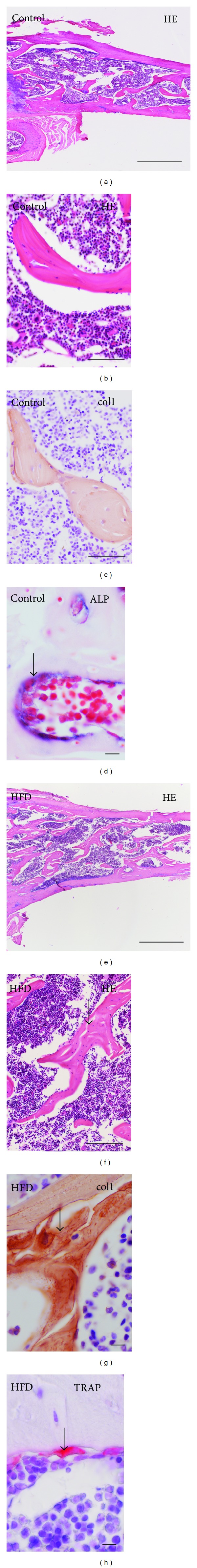
Microstructure of cancellous bone in demineralized paraffin sections. Trabeculae of control mice ((a)–(d)) exhibit a plate-like shape ((a)-(b)) whereas trabeculae of HFD mice ((e)-(f)) show a more rod-like form ((e)-(f)). The trabecular lamellae were interrupted by spaces in HFD mice (arrow, (f)). Collagen-1 immunohistochemistry (col1, (c), (g)) brought out an increase in woven bone (arrow) in (g) HFD mice in comparison to the lamellar trabeculae in (c) control animals. Changes were neither demonstrated in enzyme histochemistry of alkaline phosphatase (arrow, ALP, (d)) nor in tartrate-resistant acidic phosphatase (arrow, TRAP, (h)). Scale bar: 500 *μ*m in (a), (e); 50 *μ*m in (b), (c), (f); 20 *μ*m in (d), (g), (h).

The trabecular structure was evaluated using Epon embedded calcified semi-thin sections (Figure 3) and demineralized paraffin sections (Figure 4) that were stained routinely with hematoxylin and eosin (HE) (Figures 4(a), 4(b), 4(e), and 4(f)) or immunohistochemically labeled with an antibody against collagen-1 (Figures 4(c) and 4(g)). Semi-thin and HE sections demonstrated that the trabeculae of animals fed with HFD were smaller (Figures 3(a) and 3(b)), peaked at the ends (rod-like shape, Figures 4(b) and 4(f)), and less linked with other trabeculae (Figures 3(a) and 3(b)). The lamellar structure was often dissolved inside the trabeculae so that there was space in between the different lamellae (Figure 4(f)). In the HFD group the trabeculae contained more woven bone and less lamellar bone than in the control group as shown by collagen-1 IHC (Figures 4(c) and 4(g)). Furthermore the animals with high-fat diet exhibited less osteoid and more megakaryocytes in the bone marrow of the same samples (Figure 3(b)). However, no changes could be observed in the amount, distribution, and size of osteoclasts identified with the TRAP enzyme histochemical staining (Figure 4(h)). The ALP staining could not point out differences between both animal groups. No alterations were shown in the labeling intensity, amount, and distribution of ALP (Figure 4(c)).

### 3.4. Histomorphometry

The ratio of bone area in correlation with tissue area was measured using histomorphometry. The bone area was calculated as percentage (%) of the whole tissue. Comparing the average of the values a distinct decrease in bone was observed for all measured areas (proximal, distal femur, and vertebrae, Table 2). However, because of the high standard deviation (SD) only the values of the distal femur showed significant changes (*P* = 0.026, Table 2).

### 3.5. Real-Time RT-PCR

**Figure 5:**
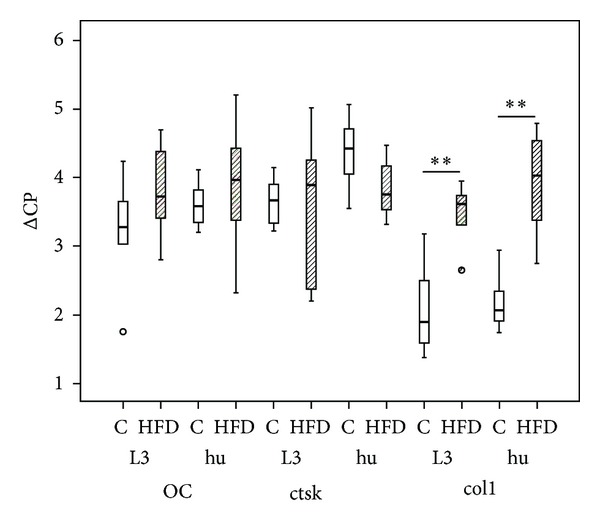
Real-time RT-PCR. The expression of collagen 1*α*1 (col1) was decreased in humerus (hu; HFD *n* = 7, C *n* = 6) and vertebrae L3 (L3; HFD *n* = 6, C *n* = 6) in HFD mice in comparison to controls (C). No regulation was found for osteocalcin (OC) and cathepsin K (ctsK) expression. Data presented as box plots with the median indicated by solid line within the box. Small circles represent data beyond 1.5x the interquartile range of the median. **P ≤ 0.01.

The expression of collagen 1*α*1 was significantly downregulated in humeri (*P* = 0.002, relative expression 0.34 ± 0.19) and vertebrae (*P* = 0.004, relative expression 0.4 ± 0.14) whereas osteocalcin and cathepsin K were not significantly changed due to HFD (Figure 5).

### 3.6. Ultrastructure

**Figure 6:**
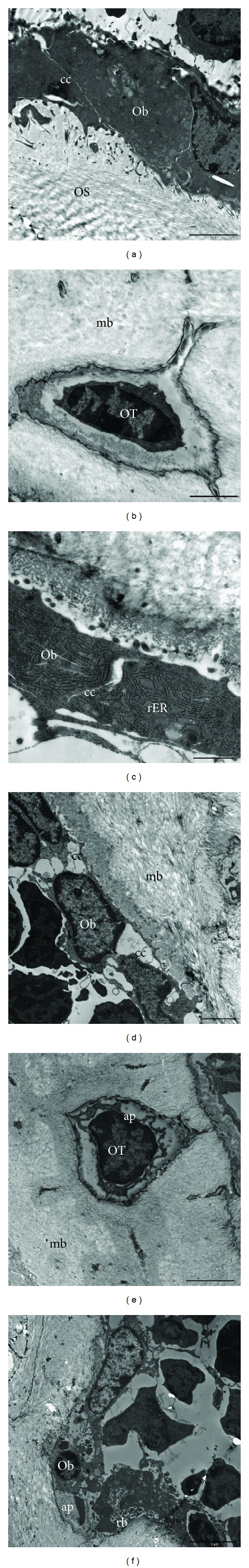
Transmission electron microscopy. In comparison to controls (a)–(c) the HFD mice (d)–(f) showed less cell-to-cell (cc) and cell-to-matrix contacts ((a), (d)). More apoptotic formations (ap) were found in osteocytes (OT, (e)) and osteoblasts (Ob, (f)) of HFD mice that also contained less rough endoplasmatic reticulum (rER) in comparison to control animals (c). mb = mineralized bone; rb = ruffled border of a osteoclast. Scale bar: 5 *μ*m in (a), (f), 2 *μ*m in (b), (d)-(e), and 1 *μ*m in (c).

TEM analysis further confirmed the presence of less osteoid in HFD mice compared to controls (Figure 6). Even if collagen fibrillae were found the striation was less distinct than in control mice. Besides, we observed a dissolving of the cell-cell and cell-matrix connections (Figure 6(d)). Spaces were formed between the single osteoblasts. In addition, osteoblasts included less rough endoplasmatic reticulum (Figure 6(d)). The membrane of osteocytes and osteoblasts was sometimes leaky and folded (Figures 6(e) and 6(f)) and more apoptotic cells were found in the HFD group.

## 4. Discussion

The presented study was conducted to analyze the bones of male mice (strain C57BL/6J) after feeding with a HFD (60% kcal from fat) for 23 weeks. The diet started at an age of 4 weeks. This animal procedure is a well-known model for induction of obesity that is used in a number of studies [15, 29–31]. Obesity is one of the most important risk factors for several musculoskeletal disorders [32]. It remains still unclear through which mechanism the adipose tissue affects the musculoskeletal system. It is known that additional load leads to functional and structural limitation of the soft structures such as tendons [33]. Furthermore overweight results in less physical activity [34] and that causes a loss of bone mass [35]. On the other hand adipose tissue secretes adipose-derived hormones and cytokines (e.g, TNF*α*, IL-1, and IL-6) leading to a low-grade systemic inflammation [12, 13, 15]. These factors are also involved in the cross talk of bone cells [14]. However, relation between obesity and bone is still controversially discussed, and therefore the aim of the present study was to investigate the bone structure of obese mice in comparison to normal animals by means of DXA, histological methods, histomorphometrical measurement, real-time RT-PCR, and transmission electron microscopy.

DXA-scan allows the calculation of BMD which in our study showed no differences between obese mice and controls getting normal chow (10% kcal from fat). A close correlation of body weight and BMD, however, was found in humans where an approximate increase in 10 kg body weight causes a 1% increase in BMD [36]. This positive correlation is stronger in women than in men and in postmenopausal than in premenopausal women. This effect could be explained by the conversion of androgens into estrogen in adipocytes and by the protective role of estrogen against osteoporosis and bone loss (reviewed in [10]). Besides, Ehrlich and Lanyon described that the biomechanical loading of the additive weight stimulates bone formation and therefore increases bone mass and BMD [37]. Contrasting studies demonstrated that the bone strengthening effects of heavy bodies were not only due to adipose tissue but also due to elevated muscle mass [38]. However, in the present study no significant alterations were measured regarding the BMD.

Changes in bone mineral density are often induced by an imbalance in the amount and activity of bone forming osteoblasts and bone resorbing osteoclasts. Enzyme histochemistry of alkaline phosphatase is a common method for testing the activity of osteoblasts [39]. ALP activity showed no alteration in bone sections from mice receiving HFD compared to control diet. Cao et al., 2009, found an upregulation of ALP positive colony forming units after culturing bone marrow stromal/osteoblastic cells of mice fed with HFD [40]. The direct comparison between these results is not possible because of the different methods and the different HFDs. In the present study we used a HFD where 60 kcal% energy as fat was given whereas the mice in Cao et al.'s [40] study got a HFD with 45 kcal% energy as fat. Hence, there could be a metabolic window were an increase in fat has positive effects on bone formation. Such a window would also explain the controversial discussion in the literature and this would be in line with the report ofNúñez et al., 2007, who described that extreme obesity reduces BMD in animals and humans [41]. Furthermore, bone stimulating effects are only found when the energy upregulation is not correlated with an increase of insulin. In patients with type II diabetes mellitus osteoblasts increased their cell division and proliferation in presence of insulin (1.2- to 1.7-fold) but the ALP activity and the production of mineralized matrix was reduced to 55% in comparison to control [39]. However, in the present study no alteration was observed in ALP activity. ALP is an enzyme that is necessary for the mineralization of the bone matrix. It is linked to the membrane of matrix vesicles via a glycosylphosphatidylinositol (GPI) anchor by means of posttranslational modification. ALP is involved in the formation of hydroxyapatite crystals within the vesicles by hydrolyzing monophosphate esters at a pH of 8–10. After the matrix vesicles are budding from the osteoblast, the hydroxyapatite crystals penetrate the vesicle membrane and fill the space between the collagen fibrils (reviewed in [42]).

Besides ALP, osteocalcin and collagen-1 are very prominent in bone. With an amount of 1% of the bone matrix, osteocalcin is the most important member of the group of noncollagenous matrix proteins. In our study we analyzed the possible regulation of osteocalcin on mRNA level where we did not find any alteration. On the protein level Cao et al. [40] could measure a significant down-regulation and therefore supposed a delay in bone formation. Besides, collagen-1 is the main component of the non-mineralized bone matrix. Using real-time RT-PCR we observed a significant down-regulation of collagen 1*α*1 mRNA in both bone types, long bone (tibia) as well as irregular bone (vertebrae) of HFD mice. Thus, on mRNA level, the formation of non-mineralized matrix is changed in our obesity model. Since the collagen builds up the skeletal structure of bone, we measured the cancellous area in relation to the whole tissue by means of histomorphometry. Our results showed a distinct downregulation in the relative trabecular area of HFD mice in comparison to the control mice. A decrease in cancellous bone mass has also been reported by other studies using HFD in mice as model for obesity [29, 30, 40]. In addition to histomorphometry used in our study Patsch et al. also used microcomputed tomography (*μ*CT) [29] and Fujita et al. and Cao et al. focused on *μ*CT and serum levels of bone markers [30, 40]. *μ*CT analysis possesses the advantage of analyzing the bone structures 3-dimensionally whereas histomorphometry is restricted to 2 dimensions. All three reports [29, 30, 40] measured a decrease in the ratio of bone volume to tissue volume and trabecular number. In addition, Patsch et al. and Cao et al. also described an increase in trabecular separation, connectivity density, and structure model index (SMI) [29, 40]. In contrast to our study the reports of Fujita et al. [30] and Patsch et al. [29] correlated these effects with the duration of the HFD. Fujita et al. analyzed animals after 4, 8, and 12 weeks of HFD. The effects of HFD on the bone structure increased with time [30]. In our study we used only a long-term HFD (23 weeks) where we could confirm the results of Patsch et al. [29] and Fujita et al. [30]. Fujita et al. [30] correlated structure of trabecular and cortical bone. Although cortical bone formation was slower in obese mice compared to controls, the periostal bone formation increased with age. Thus, they assumed that the underlying mechanism of bone loss was different at these two sites of bone [30]. Ionova-Martin et al. confirmed these results in a study with young animals receiving a HFD. When the HFD was given to adults they measured a decrease in femoral diameter, bone strength, fracture toughness, and alignment of osteocytes [31]. Our results also depict a lamellar disorganization that affects the osteocyte microarchitecture.

Beyond the bone forming osteoblast lineage, osteoclasts are accountable for a loss of bone mass. Osteoclasts are multinucleated cells with a specific endowment of enzymes and transporters that facilitate them to resorb bone [43]. In the present study osteoclasts were analyzed on mRNA level by measuring cathepsin K expression and histologically by TRAP enzyme histochemistry. No differences were observed in the osteoclast population between HFD mice and controls using both methods. Multinucleated osteoclasts with a ruffled border, sealing zone, and several vesicles in their cytoplasm were found to be located at the surface of the mineralized bone. No structural aberration or obvious difference in the amount was found in the HFD mice in comparison to the controls. Despite this, striking alterations were found for the osteoblasts. Osteoblasts are usually situated on the surface of growing bone as a closely packed layer of cells [4] that are connected to each other, to osteocytes, and to osteoid via gap junctions, connexons, and hemi-channels [44]. An unusual big space was observed between the osteoblasts among themselves and between osteoblasts and the bone surface. The TEM observations did not give information about the connections from osteoblast to the osteocytes. However the cell-to-cell and cell-to-matrix communications are important for maintaining bone homeostasis. The expression of connexin43, which is the main component of gap junctions and hemi-channels is needed for functioning of mature osteoblasts and osteocytes [45]. This report indicates that gap junctions and hemi-channels play an important role for bone cell survival. In HFD mice several apoptotic formations were observed for osteocytes and osteoblasts, and additionally, TEM analysis showed a reduced osteoid production and striation and a decrease in osteoblast endoplasmatic reticulum in HFD mice. Thus, we suspect that the reduction of cell-to-cell contacts is followed by an increase in apoptosis of bone forming cells and therefore the assembling of new bone is delayed.

In summary, the presented study demonstrated a miss-arrangement of cell-cell and cell-matrix contacts, osteoblast and trabecular structure, and collagen-1 and osteoid synthesis that altogether outlines a negative effect of obesity on bone microstructure.

## Figures and Tables

**Table 1:** Primers used for RT-PCR.

Targets	Sequence	Length (bp)	Annealing (°C)	Accession
*β*-actin				
fwd^1^	TGTTACCAACTGGGACGACA	165	58	NM_007393.3
rev^2^	GGGGTGTTGAAGGTCTCAAA			
Bglap^3^				
fwd	TTCTGCTCACTCTGCTGACC	111	58	NM_007541.2
rev	TATTGCCCTCCTGCTTGGAC			
ctsK^4^				
fwd	GAGGCGGCTATATGACCACT	119	58	NM_007802.3
rev	CTTTGCCGTGGCGTTATACA			
col1^5^				
fwd	TGGCATCCCTGGACAGCCTG	144	62	NM_007742.3
rev	ATGGGGCCAGGCACGGAAAC			

^1^Forward; ^2^reverse; ^3^bglap: gene name of osteocalcin; ^4^cathepsin K; ^5^collagen 1*α*1.

**Table 2:** Histomorphometry.

Region	Group	Bone area (%)	SD	*P* value
Proximal femur	C	21.0009	±2.0087	*P* = 0.065
	HFD	16.8641	±4.2989	

Distal femur	C	22.0639	±5.0217	*P* = 0.026
	HFD	16.7619	±1.7276	

Vertebra L2	C	23.5852	±2.657	*P* = 0.101
	HFD	19.854	±3.961	

HFD: high-fat diet, *n* = 7; C: control mice, *n* = 6.

## References

[1] Kopelman PG (2000). Obesity as a medical problem. *Nature*.

[2] Swinburn BA, Sacks G, Hall KD (2011). The global obesity pandemic: shaped by global drivers and local environments. *The Lancet*.

[3] Gregoire FM, Smas CM, Sul HS (1998). Understanding adipocyte differentiation. *Physiological Reviews*.

[4] Clarke B (2008). Normal bone anatomy and physiology. *Clinical Journal of the American Society of Nephrology*.

[5] David V, Martin A, Lafage-Proust M-H (2007). Mechanical loading down-regulates peroxisome proliferator-activated receptor *γ* in bone marrow stromal cells and favors osteoblastogenesis at the expense of adipogenesis. *Endocrinology*.

[6] Sen B, Xie Z, Case N, Ma M, Rubin C, Rubin J (2008). Mechanical strain inhibits adipogenesis in mesenchymal stem cells by stimulating a durable *β*-catenin signal. *Endocrinology*.

[7] Gimble JM, Robinson CE, Wu X (1996). Peroxisome proliferator-activated aeceptor-*γ* activation by thiazolidinediones induces adipogenesis in bone marrow stromal cells. *Molecular Pharmacology*.

[8] Lazarenko OP, Rzonca SO, Suva LJ, Lecka-Czernik B (2006). Netoglitazone is a PPAR-gamma ligand with selective effects on bone and fat. *Bone*.

[9] Tornvig L, Mosekilde L, Justesen J, Falk E, Kassem M (2001). Troglitazone treatment increases bone marrow adipose tissue volume but does not affect trabecular bone volume in mice. *Calcified Tissue International*.

[10] Cao JJ (2011). Effects of obesity on bone metabolism. *Journal of Orthopaedic Surgery and Research*.

[11] Kirchengast S, Knogler W, Hauser G (2002). Protective effect of moderate overweight on bone density of the hip joint in elderly and old Austrians. *Anthropologischer Anzeiger*.

[12] Halade GV, El Jamali A, Williams PJ, Fajardo RJ, Fernandes G (2011). Obesity-mediated inflammatory microenvironment stimulates osteoclastogenesis and bone loss in mice. *Experimental Gerontology*.

[13] Fain JN (2010). Release of inflammatory mediators by human adipose tissue is enhanced in obesity and primarily by the nonfat cells: a review. *Mediators of Inflammation*.

[14] Sims NA, Walsh NC (2012). Intercellular cross-talk among bone cells: new factors and pathways. *Current Osteoporosis Reports*.

[15] Xiao Y, Cui J, Li Y-X, Shi Y-H, Le G-W (2010). Expression of genes associated with bone resorption is increased and bone formation is decreased in mice fed a high-fat diet. *Lipids*.

[16] Compston JE, Watts NB, Chapurlat R (2011). Obesity is not protective against fracture in postmenopausal women: glow. *American Journal of Medicine*.

[17] Rana AR, Michalsky MP, Teich S, Groner JI, Caniano DA, Schuster DP (2009). Childhood obesity: a risk factor for injuries observed at a level-1 trauma center. *Journal of Pediatric Surgery*.

[18] Manias K, McCabe D, Bishop N (2006). Fractures and recurrent fractures in children; varying effects of environmental factors as well as bone size and mass. *Bone*.

[19] Taylor ED, Theim KR, Mirch MC (2006). Orthopedic complications of overweight in children and adolescents. *Pediatrics*.

[20] Ward WE, Kim S, Bruce WR (2003). A western-style diet reduces bone mass and biomechanical bone strength to a greater extent in male compared with female rats during development. *British Journal of Nutrition*.

[21] Zernicke RF, Salem GJ, Barnard RJ, Schramm E (1995). Long-term, high-fat-sucrose diet alters rat femoral neck and vertebral morphology, bone mineral content, and mechanical properties. *Bone*.

[22] Wongdee K, Krishnamra N, Charoenphandhu N (2012). Endochondral bone growth, bone calcium accretion, and bone mineral density: how are they related?. *The Journal of Physiological Sciences*.

[23] Albala C, Yáñez M, Devoto E, Sostin C, Zeballos L, Santos JL (1996). Obesity as a protective factor for postmenopausal osteoporosis. *International Journal of Obesity*.

[24] Edelstein SL, Barrett-Connor E (1993). Relation between body size and bone mineral density in elderly men and women. *American Journal of Epidemiology*.

[25] Nguyen ND, Pongchaiyakul C, Center JR, Eisman JA, Nguyen TV (2005). Abdominal fat and hip fracture risk in the elderly: the dubbo osteoporosis epidemiology study. *BMC Musculoskeletal Disorders*.

[26] Reid IR (2010). Fat and bone. *Archives of Biochemistry and Biophysics*.

[27] Cao JJ, Sun L, Gao H (2010). Diet-induced obesity alters bone remodeling leading to decreased femoral trabecular bone mass in mice. *Annals of the New York Academy of Sciences*.

[28] Dempster DW, Compston JE, Drezner MK (2013). Standardized nomenclature, symbols, and units for bone histomorphometry: a 2012 update of the report of the ASBMR Histomorphometry Nomenclature Committee. *Journal of Bone and Mineral Research*.

[29] Patsch JM, Kiefer FW, Varga P (2011). Increased bone resorption and impaired bone microarchitecture in short-term and extended high-fat diet-induced obesity. *Metabolism*.

[30] Fujita Y, Watanabe K, Maki K (2012). Serum leptin levels negatively correlate with trabecular bone mineral density in high-fat diet-induced obesity mice. *Journal of Musculoskeletal and Neuronal Interactions*.

[31] Ionova-Martin SS, Wade JM, Tang S (2011). Changes in cortical bone response to high-fat diet from adolescence to adulthood in mice. *Osteoporosis International*.

[32] Anandacoomarasamy A, Caterson I, Sambrook P, Fransen M, March L (2008). The impact of obesity on the musculoskeletal system. *International Journal of Obesity*.

[33] Wearing SC, Hennig EM, Byrne NM, Steele JR, Hills AP (2006). Musculoskeletal disorders associated with obesity: a biomechanical perspective. *Obesity Reviews*.

[34] Wearing SC, Hennig EM, Byrne NM, Steele JR, Hills AP (2006). The biomechanics of restricted movement in adult obesity. *Obesity Reviews*.

[35] Cheng ML, Gupta V (2013). Premenopausal osteoporosis. *Indian Journal of Endocrinology and Metabolism*.

[36] Gjesdal CG, Halse JI, Eide GE, Brun JG, Tell GS (2008). Impact of lean mass and fat mass on bone mineral density: the Hordaland Health Study. *Maturitas*.

[37] Ehrlich PJ, Lanyon LE (2002). Mechanical strain and bone cell function: a review. *Osteoporosis International*.

[38] Zhao L-J, Jiang H, Papasian CJ (2008). Correlation of obesity and osteoporosis: effect of fat mass on the determination of osteoporosis. *Journal of Bone and Mineral Research*.

[39] Freude T, Braun KF, Haug A (2012). Hyperinsulinemia reduces osteoblast activity in vitro via upregulation of TGF-beta. *Journal of Molecular Medicine*.

[40] Cao JJ, Gregoire BR, Gao H (2009). High-fat diet decreases cancellous bone mass but has no effect on cortical bone mass in the tibia in mice. *Bone*.

[41] Núñez NP, Carpenter CL, Perkins SN (2007). Extreme obesity reduces bone mineral density: complementary evidence from mice and women. *Obesity*.

[42] Orimo H (2010). The mechanism of mineralization and the role of alkaline phosphatase in health and disease. *Journal of Nippon Medical School*.

[43] Teitelbaum SL (2007). Osteoclasts: what do they do and how do they do it?. *American Journal of Pathology*.

[44] Batra N, Kar R, Jiang JX (2011). Gap junctions and hemichannels in signal transmission, function and development of bone. *Biochimica et Biophysica Acta*.

[45] Plotkin LI, Bellido T (2013). Beyond gap junctions: connexin43 and bone cell signaling. *Bone*.

